# Association of both depressive symptoms scores and specific depressive symptoms with all-cause and cardiovascular disease mortality

**DOI:** 10.1186/s12991-024-00509-x

**Published:** 2024-07-15

**Authors:** Tao Liu, Lili Wang, Zhijian Zhu, Bing Wang, Zhigang Lu, Yesheng Pan, Lifang Sun

**Affiliations:** 1 Department of Cardiology, Jinshan District Central Hospital Affiliated to Shanghai University of Medicine and Health Sciences, Shanghai, China; 2 Department of Cardiology, The Second Affiliated Hospital of Xuzhou Medical University, Xuzhou, China; 3 Department of Cardiology, Shanghai Jiao Tong University Affiliated Sixth People’s Hospital, Shanghai, China

**Keywords:** Depressive symptoms scores, Specific depressive symptoms, All-cause mortality, CVD mortality, NHANES

## Abstract

**Background:**

The presence of depression related to an increased risk of all-cause and cardiovascular disease (CVD) mortality has been reported. However, studies conducted on certain specific depressive symptoms are scarce. Our purpose was to assess the effect of both depressive symptoms scores and certain specific depressive symptoms on all-cause and CVD mortality.

**Methods:**

In the present cohort study, all participants, aged 18 years or older, were enrolled in the National Health and Nutrition Examination Survey (NHANES) from 2005 to 2014. Depressive symptoms score was assessed using the validated 9-item Patient Health Questionnaire Depression Scale (PHQ-9), which ranges from 0 to 27, with a PHQ-9 score ≥ 10 diagnosed as depression. The outcome events were all-cause and CVD mortality, which were followed up from 2005 to 2014. The associations of both depressive symptoms score and certain specific depressive symptoms with all-cause and CVD mortality were examined by weighted multivariable proportional hazards models.

**Results:**

A total of 26,028 participants aged ≥ 18 years were included in the statistical analysis, including 12,813 (49.2%) males and 13,215 (50.8%) females, with a mean (SD) age of 47.34 (18.86) years. During the 9.32 (3.20) years of mean (SD) follow-up, 3261 deaths were recorded, of which 826 were cardiovascular deaths. All-cause mortality was 16.87/1000 person-years in subjects with depression. In terms of CVD mortality, these figures were 4.53/1000 person-years. In the full model (model 3), elevated depressive symptoms scores were independently associated with an increased risk of all-cause mortality (Highest depression symptom score group: adjusted hazard ratio, 1.63; 95% CI 1.44–1.85) and CVD mortality (Highest depression symptom score group: adjusted hazard ratio, 1.73; 95% CI 1.34–2.24). All 9 specific depressive symptoms that make up the PHQ-9 were related to an increased risk of all-cause mortality. However, only 3 symptoms, including trouble sleeping or sleeping too much, poor appetite or overeating, and suicidal ideation, were no significantly associated with an increased risk of CVD mortality.

**Conclusions:**

The elevated depressive symptoms scores were strongly associated with an increased risk of all-cause and CVD mortality in US adults. Furthermore, all 9 specific depressive symptoms were associated with high all-cause mortality. However, trouble sleeping or sleeping too much, poor appetite or overeating, and suicidal ideation might not increase the risk of CVD mortality.

**Supplementary Information:**

The online version contains supplementary material available at 10.1186/s12991-024-00509-x.

## Introduction

Depression is one of the most common mental disorders in the world, and depressive symptoms are also common in the population [1]. Like much of the world, the prevalence of Americans is increasing. The incidence of depression has become an increasingly important public-health priority [2]. Studies have shown that the lifetime and 12-month prevalence are 20.6% and about 10.4%, respectively, and the prevalence of women (26.1%) is higher than that of men (14.7%) [3]. Notably, depressive symptoms have been associated with death from all causes, such as all-cause mortality, cardiovascular disease (CVD) mortality, and cancer mortality [3–6]. The higher the severity of depressive symptoms, the greater the risk of death [7]. Therefore, to reduce mortality, understanding the association between depressive symptoms and mortality is important.

Currently, depressive symptoms are mainly assessed by some rating scales, such as the Center for Epidemiological Studies Depression Scale (CES-D) [8] and the Geriatric Depression Scale [9]. These scales identify depression disorder with established and validated cut-off points, and although these scales cannot diagnose major depressive disorders, they have been widely applied in research and daily practice. Most previous studies [5, 7, 10–12] examining the association between depressive symptoms and risk of death have only analyzed the presence or absence of depression or the total score on the scales. Although analyzing specific depression symptoms is also essential [13], there is a lack of evidence to support an association between specific depressive symptoms and mortality. Sheida Zolfaghari et al. found that sleep disorders (one form of depressive symptom) were associated with an increased risk of death, and that these effects differed by gender [14]. Quanhe Yang et al. reported that poor appetite or overeating was independently associated with both all-cause and cardiovascular mortality [11]. However, there are very few such studies, and the relationship between each specific depressive symptom and mortality is unclear. Due to the heterogeneity of depressive symptoms and important differences between depressed individuals, therefore, studies on specific depressive symptoms associated with mortality could provide new ideas for individualized treatment of patients with depression disorder.

Until now, the impact of both depressive symptoms scores and specific depressive symptoms on all-cause and CVD mortality has remained unknown, which prompted us to conduct this study. We assumed that depressive symptoms scores and certain specific depressive symptoms would be associated with an increased risk of all-cause and CVD mortality.

## Methods

### Study population

This study is a cohort study with data from the publicly available National Health and Nutrition Examination Survey (NHANES) database. Conducted every 2 years, the health survey program uses a complex, multicenter, stratified sampling design with data collected from a nationally representative sample of U.S. civilians to monitor the health and nutritional status of adults and children across the United States [15]. Details of the survey design and the method are available on the NHANES website (https://www.cdc.gov/nchs/nhanes/index.htm).

This study initially included participants aged ≥ 18 years from five survey cycles in the period 2005–2014. 4211 participants with incomplete information on depressive symptoms were excluded, and 26,028 participants were included in the final analysis. What is more, all participants included in the final analysis were effectively followed up. The project was approved by the Research Ethics Review Board of the National Center for Health Statistics (Protocol #2005-06 and #2011-17) and was in accordance with the Strengthening the Reporting of Observational Studies in Epidemiology (STROBE) reporting guidelines (https://www.cdc.gov/nchs/nhanes/irba98.htm). Informed consent has been obtained from all participants for the NHANES data. Due to the free availability of the NHANES database, the present study does not require additional ethical review and approval.

### Evaluation of depressive symptoms

The depressive symptoms of the study subjects were assessed according to the 9-item Patient Health Questionnaire Depression Scale (PHQ-9) [16, 17]. The PHQ-9 is an internationally validated screening and diagnostic tool for depressive symptoms that assesses the frequency and severity of various depressive symptoms over the past two weeks [18]. PHQ-9 is composed of 9 items: (1) have little interest in doing things; (2) feeling down, depressed, or hopeless; (3) trouble sleeping or sleeping too much; (4) feeling tired or having little energy; (5) poor appetite or overeating; (6) feeling bad about yourself; (7) trouble concentrating on things; (8) moving or speaking slowly or too fast; and (9) thought you would be better off dead. Each question was scored as 0 (not at all); 1 (several days); 2 (more than half the days); and 3 (nearly every day) [16]. The total score was calculated by summing the scores for each item and ranged from 0 to 27, with depressive symptom severity increasing with the score. Based on the diagnostic criteria for depression in the NHANES, participants with scores ≥ 10 were considered to suffer from depression [19].

### Assessment of outcome status

The primary outcome indicators for this study consisted of all-cause mortality and CVD mortality. The survival status of participants was tracked through death files, which were collected by the NHANES-linked National Death Index (NDI) (https://www.cdc.gov/nchs/data-linkage/mortality-public.htm#). The files provide details of follow-up visits from the date of the interview to December 31, 2015. The specific causes of death are classified according to the International Classification of Diseases guidelines, 10th revision (ICD-10). All-cause mortality was ascribed to death from any cause. CVD mortality was determined as death due to cardiovascular disease (ICD-10 codes: I00-I09, I11, I13, and I20-I51) or cerebrovascular disease (ICD-10 codes: I60-I69).

### Ascertainment of covariates

Demographic information was acquired through interviews and questionnaires: age, sex, race (Mexican, Non-Hispanic white, Non-Hispanic black, and other), and education level (Less than high school, High school, and above high school). A physical examination was conducted by professional medical examiners to obtain relevant index data: waist, systolic blood pressure (SBP), and diastolic blood pressure (DBP). Personal history included self-reported smoking status and drinking status (Never, Former, or Current). Medical Conditions consist of Congestive heart failure, Coronary heart disease, Hypertension, Hyperlipidemia, Diabetes, Chronic kidney disease (CKD), and Stroke. Hypertension was defined as meeting one of the following criteria: (1) SBP ≥ 140 mmHg or DBP ≥ 90 mmHg measured at baseline; (2) taking antihypertensive medication; (3) a self-reported history of hypertension [20]. Diabetes was characterized by fasting blood glucose ≥ 7.0 mmol/L or hemoglobin ≥ 6.5%, a self-reported history of diabetes, or being on anti-diabetic medication [21]. Hyperlipidemia was described as one of the following conditions: (1) triglycerides ≥ 150 mg/dL; (2) total cholesterol ≥ 200 mg/dL; (3) low-density lipoprotein-cholesterol ≥ 130 mg/dL; (4) high-density lipoprotein-cholesterol < 40 mg/dL (male) or < 50 mg/dL (female); and (5) use of cholesterol-lowering drugs [22]. An estimated glomerular filtration rate (eGFR) of < 60 mL/min/1.73 m^2^ was classified as CKD [23]. The eGFR was measured with the Scr formula recommended by the Chronic Kidney Disease Epidemiology Collaborative Group in 2009 [24]. The details of all data could be found at https://www.cdc.gov/nchs/nhanes/.

### Statistical analyses

The data were weighted with the Taylor linearization method according to the design of NHANES multi-stage probability sampling. Continuous variables were expressed as mean (standard deviation, SD), and categorical variables were displayed as frequency (percentage). Baseline characteristics of continuous and categorical variables were compared between groups by the T-test and the Chi-square test, respectively. The detailed missing data was described in Supplementary Table 1.

The follow-up period ranged from the survey date at baseline (2005–2014) to the time of death, end of follow-up, or loss of follow-up, depending on each participant. All-cause and CVD mortality rates for each 1000 person-years were computed based on depressive symptoms. Cox proportional hazard regression models were performed to explore the association of both depressive symptoms scores and specific depressive symptoms with all-cause and CVD mortality, and the results were expressed as hazard ratios (HRs) and 95% confidence intervals (CIs). There were three models: Model 1 adjusted for sex, age, and race; Model 2 adjusted for age, sex, race, education level, smoking status, drinking status, waist, and SBP; and Model 3 made additional adjustments for congestive heart failure, coronary heart disease, hypertension, hyperlipidemia, diabetes, chronic kidney disease, and stroke based on Model 2. In addition, nine specific depressive symptoms were simultaneously added to Model 3 for analysis.

Furthermore, participants were divided into 5 groups (Q1: 0–0, Q2: 0–1, Q3: 1–2, Q4: 2–4, Q5: 5–27) based on quintiles of total depressive symptoms scores. The correlations between depressive symptoms scores and mortality were examined using Cox proportional risk regression models (models 1–3), with the Q1 group as the reference group. Meanwhile, restricted cubic spline (RCS) plots were performed to show the dose–response relationship between total depressive symptoms scores and all-cause and CVD mortality. Kaplan–Meier curves and log-rank tests were conducted to describe the association between depressive symptom scores and the corresponding survival rates. Stratification by baseline characteristics (age, sex, and race, education level, smoking status, drinking status, waist, congestive heart failure, coronary heart disease, hypertension, hyperlipidemia, diabetes, chronic kidney disease, and stroke) was performed to explore whether the association between depressive symptoms scores (Q1-Q5) and the risk of all-cause and CVD mortality differs in different subgroups. The *P*-value for interaction was applied to reflect whether interactions occurred, and the *P*-value for trend was applied to examine the trend of linear change.

Sensitivity analyses were performed: participants with missing values (n = 4476) were excluded, and the Cox proportional risk regression analysis was repeated for participants with complete data (n = 21,552). All tests were performed using two-sided tests a statistical significance level of 0.05. Statistical analyses were performed through R 4.2.1 software.

## Results

The baseline characteristics of the study population are shown in Table 1. A total of 26,028 participants aged ≥ 18 years were included in the statistical analysis, including 12,813 (49.2%) males and 13,215 (50.8%) females, with a mean (SD) age of 47.34 (18.86) years. At baseline, 2291 (8.8%) participants were diagnosed with depression disorder (total score of PHQ-9 ≥ 10). Compared with the non-depressed population, the depressed population were more likely to be female (64.3% vs. 49.5%; *P*-value < 0.001), have less than high school education (38.5% vs. 25.3%; *P*-value < 0.001), be smokers (37.8% vs 19.6%; *P*-value < 0.001), be former drinkers (24.7% vs 18.1%; *P*-value < 0.001), have combination of Congestive heart failure (6.7% vs 2.8%; *P*-value < 0.001), Coronary heart disease (6.4% vs 3.7%; *P*-value < 0.001), Hypertension (49.6% vs 38.8%; *P*-value < 0.001), Hyperlipidemia (73.9% vs 67.7%; *P*-value < 0.001), Diabetes (25.6% vs 16.4%; *P*-value < 0.001), Chronic kidney disease (21.5% vs 17.6%; *P*-value < 0.001), and Stroke (7.9% vs 3.5%; *P*-value < 0.001), and have higher levels of DBP (mean [SD], 70.23 [12.49] vs 69.26 [12.12]; *P*-value < 0.001) and waist (mean [SD], 101.77 [18.34] vs 97.97 [16.19]; *P*-value < 0.001). There were no significant differences in age, race, and SBP between the two groups. In addition, the baseline characteristics of participants with complete data information (n = 21,552) are shown in Supplementary Table 2, and similar baseline differences were found.

**Table 1:** Baseline characteristics of 26,028 participants according to depressive symptoms status

Characteristics	Participants, No. (%)			*P*-value^b^
	Overall	Depressive symptoms status		
	N = 26,028	No (N = 23,737)	Yes (N = 2291)^a^	
Age, mean (SD), years	47.34 (18.86)	47.36 (19.04)	47.18 (16.80)	0.654
Female (%)	13,215 (50.8)	11,741 (49.5)	1474 (64.3)	< 0.001
Ethnicity				0.059
White	11,669 (44.8)	10,699 (45.1)	970 (42.3)	
Black	5653 (21.7)	5126 (21.6)	527 (23.0)	
Mexican	4243 (16.3)	3869 (16.3)	374 (16.3)	
Other	4463 (17.1)	4043 (17.0)	420 (18.3)	
Education level^c^				< 0.001
< High school diploma	6872 (26.4)	5990 (25.3)	882 (38.5)	
High school diploma	6186 (23.8)	5627 (23.7)	559 (24.4)	
> High school diploma	12,950 (49.8)	12,100 (51.0)	850 (37.1)	
Smoking status^c^				< 0.001
Never	13,441 (54.4)	12,543 (55.8)	898 (40.7)	
Former	6011 (24.3)	5538 (24.6)	473 (21.5)	
Current	5242 (21.2)	4409 (19.6)	833 (37.8)	
Drinking status^c^				< 0.001
Never	3672 (14.7)	3366 (14.8)	306 (13.8)	
Former	4661 (18.7)	4114 (18.1)	547 (24.7)	
Current	16,607 (66.6)	15,245 (67.1)	1362 (61.5)	
Waist, mean (SD), cm	98.30 (16.42)	97.97 (16.19)	101.77 (18.34)	< 0.001
Blood pressure, mean (SD), mmHg^c^				
Systolic	122.39 (18.25)	122.42 (18.12)	122.12 (19.60)	0.461
Diastolic	69.35 (12.16)	69.26 (12.12)	70.23 (12.49)	< 0.001
History of comorbidities				
Congestive heart failure^c^	767 (3.2)	621 (2.8)	146 (6.7)	< 0.001
Coronary heart disease^c^	961 (4.0)	823 (3.7)	138 (6.4)	< 0.001
Hypertension^c^	10,342 (39.7)	9205 (38.8)	1137 (49.6)	< 0.001
Hyperlipidemia	17,771 (68.3)	16,077 (67.7)	1694 (73.9)	< 0.001
Diabetes^c^	4393 (17.2)	3819 (16.4)	574 (25.6)	< 0.001
Chronic kidney disease^c^	4445 (18.0)	3977 (17.6)	468 (21.5)	< 0.001
Stroke^c^	950 (3.9)	778 (3.5)	172 (7.9)	< 0.001

^a^Defined as a score of 10 or higher on the nine-item center for the Patient Health Questionnaire in clinical studies

^b^*P*-value was based on T test or χ^2^

^c^Missing data: Education level (20 of 26,028 [0.08%]), Smoking status (1334 of 26,028 [5.13%]), Drinking status (1088 of 26,028 [4.18%]), Waist (753 of 26,028 [2.89%]), Systolic blood pressure (571 of 26,028 [2.19%]), Diastolic blood pressure (667 of 26,028 [2.56%]), Congestive heart failure (1703 of 26,028 [6.54%]), Coronary heart disease (1728 of 26,028 [6.64%]), Hypertension (3 of 26,028 [0.01%]), Diabetes (553 of 26,028 [2.12%]), Chronic kidney disease Diabetes (1295 of 26,028 [4.98%]), Stroke (1668 of 26,028 [6.41%])

**Fig. 1:**
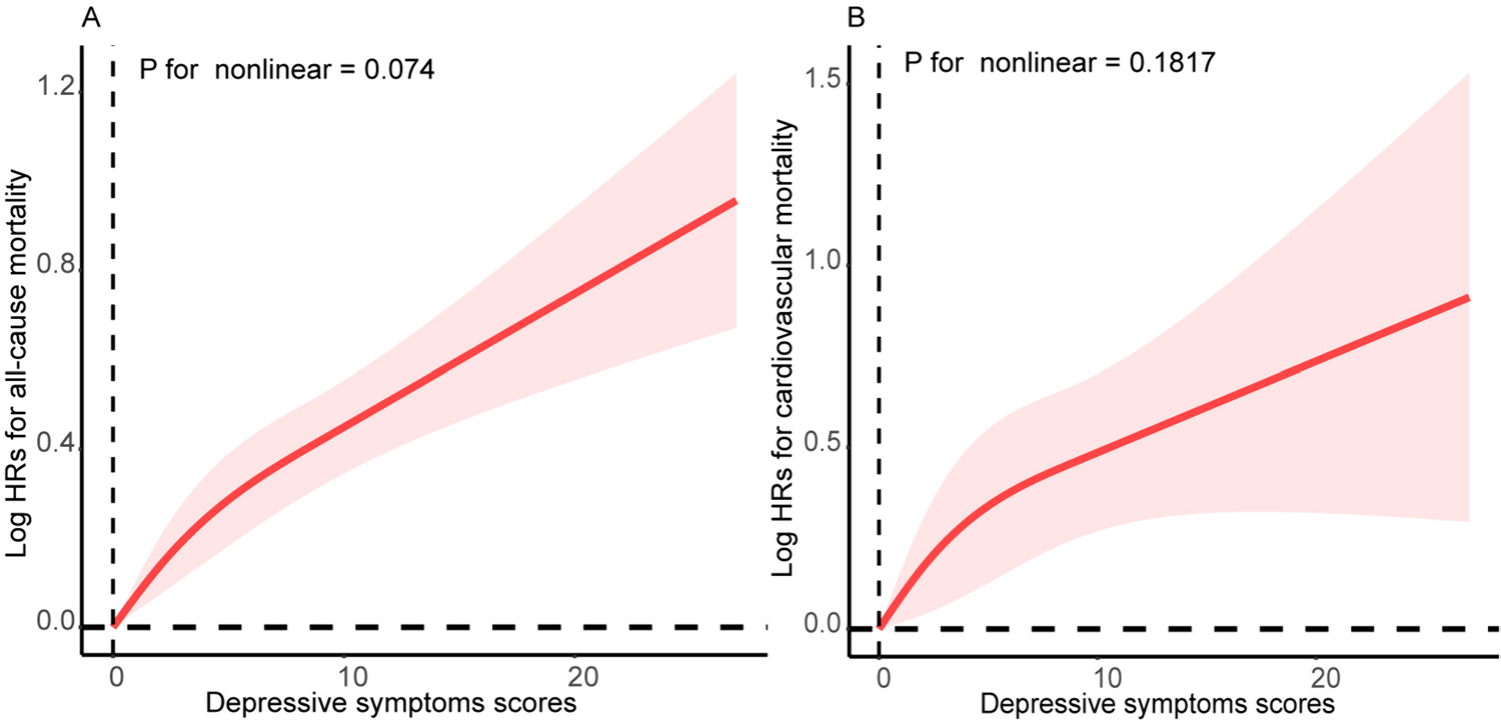
Adjusted restricted cubic spline curve for association of depressive symptoms score with incidence of all-cause mortality (**A**) and cardiovascular mortality (**B**)

**Fig. 2:**
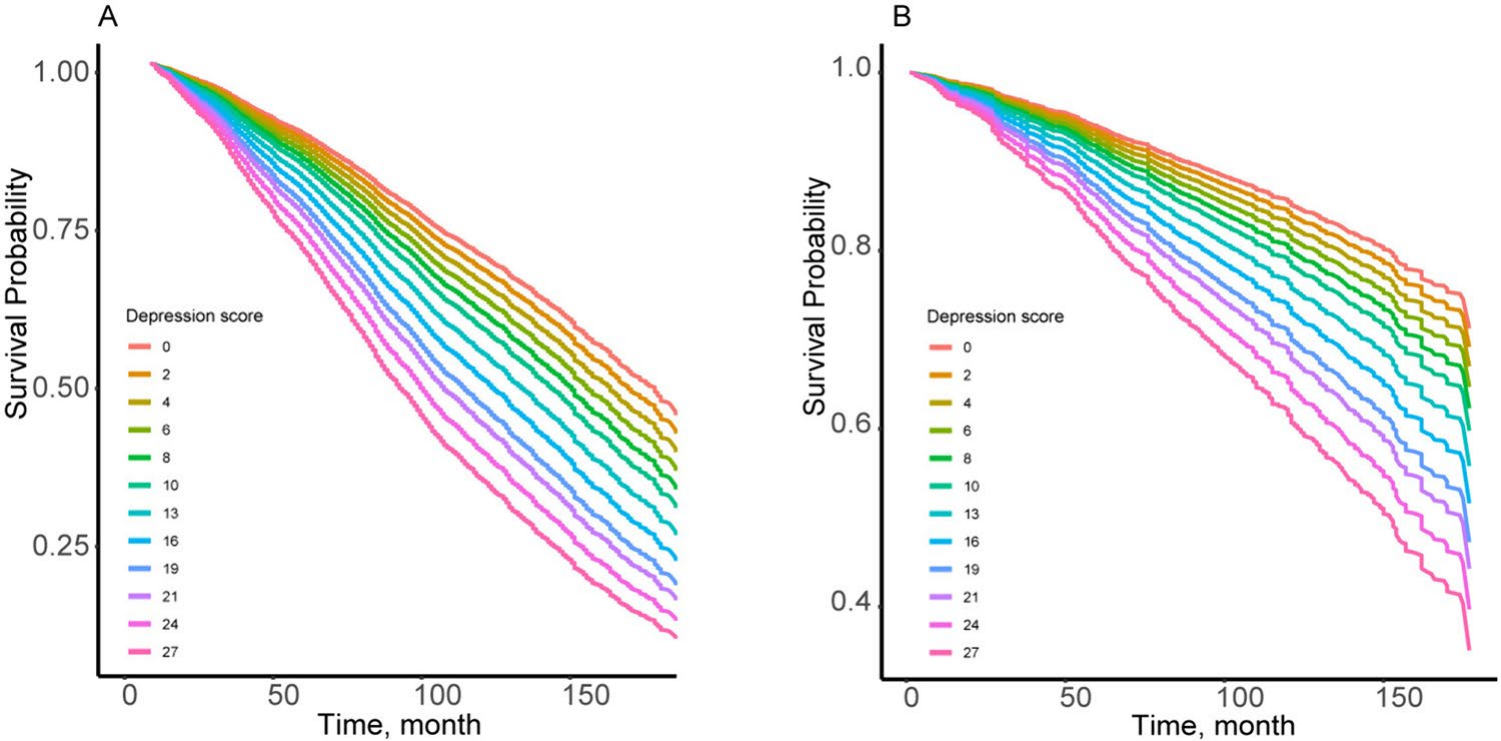
Kaplan–Meier survival curve for association of depressive symptoms score with incidence of all-cause mortality (**A**) and cardiovascular mortality (**B**)

Between the follow-up periods of 2005 and 2014, 3261 deaths were recorded, of which 826 were CVD deaths. All-cause mortality was 16.87/1000 person-years in subjects with depression and 13.13/1000 person-years in subjects without depression. In terms of CVD mortality, these figures were 4.53/1000 person-years and 3.30/1000 person-years, respectively. Table 2 displays the relationships between depression with all-cause and CVD mortality in models 1, 2, and 3. After full adjustment for potential confounders (Model 3), the presence of depression increased the risk of all-cause mortality by 51% (HR, 1.51; 95% CI 1.30–1.76; *P*-value < 0.001) and the risk of CVD mortality by 76% (HR, 1.76; 95% CI 1.36–2.26; *P*-value < 0.001). Participants with missing values were excluded, and consistent results were gained when the remaining complete data were re-analyzed (Supplementary Table 3). Participants were divided into five groups according to the quintiles of the total PHQ-9 score, as shown in Table 2. Participants in the Q5 group had a significantly higher incidence of all-cause mortality (HR, 1.63; 95% CI 1.44–1.85; *P*-value < 0.001) compared to the reference group (Q1). Similar results were obtained for CVD mortality using the same statistical treatment (HR, 1.73; 95% CI 1.34–2.24; *P*-value < 0.001). RCS plots demonstrated that there were linear relationships between total depressive symptom scores with all-cause (*P*-value for nonlinear = 0.074) and CVD mortality (*P*-value for nonlinear = 0.182). All-cause and CVD mortality tended to increase in the same direction as the total depressive symptom score, as shown in Fig. 1A and B. Consistent results were obtained in Kaplan–Meier survival curves. With the extension of the follow-up period, the survival rate of each depressive symptom score group tended to decrease, either in the all-cause mortality group (Fig. 2A**)** or the CVD mortality group (Fig. 2B**)**. In addition, the survival rate of the low-score group was always higher than that of the high-score group at the same time period.

**Table 2:** Association of both depression and depressive symptoms scores with Incidence of all-cause mortality and cardiovascular mortality

Outcome	Case, No.	Incidence rate, per 1000 person-years	HR (95% CI)		
			Model 1^a^	Model 2^b^	Model 3^c^
All-cause mortality					
Depression					
No	2915	13.13	1 [Reference]	1 [Reference]	1 [Reference]
Yes^d^	346	16.87	2.11 (1.83–2.43)***	1.70 (1.47–1.97)***	1.51 (1.30–1.76)***
Depressive symptoms scores^e^, quintitle					
1(0–0)	1040	12.89	1 [Reference]	1 [Reference]	1 [Reference]
2 (0–1)	439	11.86	1.09 (0.93–1.27)	1.10 (0.94–1.28)	1.08 (0.91–1.29)
3 (1–2)	373	12.74	1.22 (1.04–1.43)*	1.19 (1.03–1.39)*	1.15 (0.99–1.33)
4 (2–4)	631	12.90	1.30 (1.12–1.50)***	1.24 (1.08–1.43)**	1.20 (1.04–1.39)*
5 (5–27)	778	16.69	2.20 (1.92–2.51)***	1.87 (1.65–2.12)***	1.63 (1.44–1.85)***
Cardiovascular mortality					
Depression					
No	733	3.30	1 [Reference]	1 [Reference]	1 [Reference]
Yes^d^	93	4.53	2.60 (2.05–3.31)***	2.12 (1.66–2.72)***	1.76 (1.36–2.26)***
Depressive symptoms scores^e^, quintitle					
1(0–0)	247	3.06	1 [Reference]	1 [Reference]	1 [Reference]
2 (0–1)	115	3.11	1.22 (0.94–1.57)	1.25 (0.96–1.62)	1.20 (0.89–1.60)
3 (1–2)	102	3.48	1.40 (1.09–1.80)**	1.37 (1.06–1.75)*	1.27 (0.99–1.63)
4 (2–4)	149	3.05	1.37 (1.08–1.73)**	1.31 (1.03–1.66)*	1.22 (0.95–1.56)
5 (5–27)	213	4.57	2.60 (2.09–3.23)***	2.21 (1.77–2.78)***	1.73 (1.34–2.24)***

HR, hazard ratio

^*^P-value < 0.05; **P-value < 0.01; ***P-value < 0.001

^a^Model 1: Age, sex, and ethnicity were adjusted

^b^Model 2: Model 1 plus education level, smoking status, drinking status, waist, and systolic blood pressure were adjusted

^c^Model 3: Model 2 plus congestive heart failure, coronary heart disease, hypertension, hyperlipidemia, diabetes, chronic kidney disease, and stroke were adjusted

^d^Defined as a score of 10 or higher on the nine-item center for the Patient Health Questionnaire (PHQ-9) in clinical studies

^e^Measured by the PHQ-9, and the range of PHQ-9 is between 0 and 27 with the highest score indicating the highest risk of depressive symptoms

The correlations of specific depressive symptoms with all-cause mortality and CVD mortality were explored in Table 3. All nine items in the PHQ-9 were independently associated with all-cause mortality after full adjustment for confounders: Uninterested in things (adjusted HR, 1.21; 95% CI 1.14–1.28; *P*-value < 0.001), Feeling down (adjusted HR, 1.19; 95% CI 1.12–1.26; *P*-value < 0.001), Sleep problems (adjusted HR, 1.07; 95% CI 1.02–1.12; *P*-value < 0.001), Tiredness (adjusted HR, 1.18; 95% CI 1.12–1.24; *P*-value < 0.01), Change of appetite (adjusted HR, 1.15; 95% CI 1.09–1.22; *P*-value < 0.001), Feeling bad about yourself (adjusted HR, 1.14; 95% CI 1.08–1.22; *P*-value < 0.001), Difficulty in concentration (adjusted HR, 1.15; 95% CI 1.08–1.23; *P*-value < 0.001), Change in speed of action (adjusted HR, 1.18; 95% CI 1.09–1.29; *P*-value < 0.001), Suicidal ideation (adjusted HR, 1.26; 95% CI 1.10–1.44; *P*-value < 0.001). Similarly, six items were significantly associated with CVD mortality, and the details are shown in Table 3.

**Table 3:** Association of specific depressive symptoms with incidence of all-cause mortality and cardiovascular mortality

Items^a^	Symptoms, No. (%)	HR (95% CI)^b^	Cardiovascular mortality
		All-cause mortality	
Have little interest in doing things	6363 (24.45)	1.21 (1.14–1.28)***	1.24 (1.12–1.38)***
Feeling down, depressed, or hopeless	6309 (24.24)	1.19 (1.12–1.26)***	1.25 (1.12–1.40)***
Trouble sleeping or sleeping too much	9807 (37.68)	1.07 (1.02–1.12)**	1.05 (0.96–1.15)
Feeling tired or having little energy	12,818 (49.25)	1.18 (1.12–1.24)***	1.21 (1.10–1.32)***
Poor appetite or overeating	6281 (24.13)	1.15 (1.09–1.22)***	1.11 (0.99–1.25)
Feeling bad about yourself	4462 (17.14)	1.14 (1.08–1.22)***	1.14 (1.00–1.30)*
Trouble concentrating on things	4458 (17.13)	1.15 (1.08–1.23)***	1.13 (1.00–1.28)*
Moving or speaking slowly or too fast	2895 (11.12)	1.18 (1.09–1.29)***	1.15 (1.01–1.31)*
Thought you would be better off dead	983 (3.78)	1.26 (1.10–1.44)***	1.12 (0.89–1.40)

HR, hazard ratio

^a^Assessed by the nine-item center for the Patient Health Questionnaire in clinical studies

^b^Model were all adjusted for age, sex, and ethnicity, education level, smoking status, drinking status, waist, and systolic blood pressure, congestive heart failure, coronary heart disease, hypertension, hyperlipidemia, diabetes, chronic kidney disease, and stroke

^*^P-value < 0.05; **P-value < 0.01; ***P-value < 0.001

The stratified Cox proportional risk regression analyses were performed to investigate whether the associations between the severity of depressive symptoms score and mortality were influenced by confounding factors, and the results are summarized in Table 4. There were significant interactions between age (*P*-value for interaction = 0.004) and drinking status (*P*-value for interaction = 0.01) on the association between depressive symptoms score and all-cause mortality. In different age subgroups, the positive association between depressive symptoms score and all-cause mortality was stronger in individuals ≤ 65 years (adjusted HR, 1.59; 95% CI 1.26–1.99) compared to those > 65 years (adjusted HR, 1.42; 95% CI 1.22–1.65). In subgroups with different statuses, the positive association between depressive symptoms score and all-cause mortality was more prominent in former drinkers (adjusted HR, 1.72; 95% CI 1.42–2.07) compared with never drinkers (adjusted HR, 1.63; 95% CI 1.15–2.32) and current drinkers (adjusted HR, 1.57; 95% CI 1.32–1.88). There was no significant interaction between depressive symptom score and CVD mortality in any subgroup (all *P*-values for interaction > 0.05).

**Table 4:** Association of depressive symptoms scores with incidence of all-cause mortality and cardiovascular mortality stratified by different factors

Factors	Depressive symptoms scores^a^, quintitle HRs (95% CI)					*P*-value for trend	*P*-value for interaction
	1(0–0)	2 (0–1)	3 (1–2)	4 (2–4)	5 (5–27)		
All-cause mortality^b^							
Age, years old							< 0.001
< 65	1 [Reference]	0.95 (0.68–1.32)	1.12 (0.85–1.50)	1.06 (0.81–1.38)	1.59 (1.26–1.99)	< 0.001	
≥ 65	1 [Reference]	1.10 (0.91–1.32)	1.10 (0.93–1.30)	1.21 (1.03–1.42)	1.42 (1.22–1.65)	< 0.0001	
Sex							0.31
Female	1 [Reference]	1.04 (0.83–1.31)	1.06 (0.84–1.34)	1.16 (0.94–1.42)	1.44 (1.20–1.74)	< 0.001	
Male	1 [Reference]	1.11 (0.90–1.37)	1.24 (1.02–1.52)	1.21 (1.00–1.46)	1.80 (1.52–2.13)	< 0.0001	
Ethnicity							0.47
White	1 [Reference]	1.12 (0.92–1.36)	1.12 (0.94–1.33)	1.18 (1.00–1.38)	1.64 (1.42–1.90)	< 0.0001	
Black	1 [Reference]	0.88 (0.69–1.13)	1.25 (0.99–1.58)	1.17 (0.94–1.46)	1.62 (1.30–2.03)	< 0.0001	
Mexican	1 [Reference]	0.76 (0.39–1.48)	1.09 (0.61–1.93)	1.42 (0.95–2.12)	1.15 (0.73–1.81)	0.230	
Other	1 [Reference]	1.09 (0.64–1.86)	1.36 (0.72–2.55)	1.46 (0.89–2.40)	1.93 (1.25–2.98)	0.003	
Education level							0.04
< High school diploma	1 [Reference]	0.93 (0.71–1.21)	1.26 (0.99–1.62)	1.05 (0.83–1.33)	1.42 (1.20–1.69)	< 0.001	
High school diploma	1 [Reference]	1.03 (0.74–1.43)	1.09 (0.81–1.47)	1.31 (1.00–1.70)	1.53 (1.16–2.02)	0.001	
> High school diploma	1 [Reference]	1.18 (0.94–1.49)	1.13 (0.92–1.38)	1.20 (0.96–1.50)	1.85 (1.51–2.28)	< 0.0001	
Smoking status							0.13
Never	1 [Reference]	0.94 (0.76–1.16)	1.22 (0.97–1.54)	1.16 (0.94–1.43)	1.67 (1.31–2.12)	< 0.0001	
Former	1 [Reference]	1.24 (0.99–1.56)	1.14 (0.91–1.43)	1.15 (0.96–1.38)	1.40 (1.15–1.70)	0.002	
Current	1 [Reference]	1.02 (0.71–1.45)	1.02 (0.68–1.53)	1.30 (0.91–1.86)	1.82 (1.38–2.40)	< 0.0001	
Drinking status							0.01
Never	1 [Reference]	1.09 (0.82–1.44)	1.45 (1.05–2.00)	1.66 (1.17–2.36)	1.63 (1.15–2.32)	0.001	
Former	1 [Reference]	1.27 (0.99–1.63)	1.13 (0.89–1.43)	1.40 (1.09–1.80)	1.72 (1.42–2.07)	< 0.0001	
Current	1 [Reference]	0.99 (0.77–1.27)	1.09 (0.85–1.41)	0.96 (0.80–1.16)	1.57 (1.32–1.88)	< 0.001	
Coronary heart disease							0.10
No	1 [Reference]	1.05 (0.88–1.25)	1.09 (0.91–1.31)	1.18 (1.01–1.37)	1.65 (1.40–1.93)	< 0.0001	
Yes	1 [Reference]	1.38 (0.89–2.16)	1.58 (1.09–2.30)	1.35 (1.00–1.83)	1.52 (1.06–2.18)	0.010	
Congestive heart failure							0.18
No	1 [Reference]	1.06 (0.88–1.26)	1.15 (0.99–1.34)	1.17 (1.01–1.35)	1.65 (1.43–1.91)	< 0.0001	
Yes	1 [Reference]	1.09 (0.68–1.77)	1.18 (0.78–1.80)	1.28 (0.98–1.67)	1.38 (0.95–1.99)	0.040	
Chronic kidney disease							0.47
No	1 [Reference]	1.00 (0.81–1.24)	1.21 (0.93–1.57)	1.17 (0.95–1.46)	1.53 (1.26–1.86)	< 0.001	
Yes	1 [Reference]	1.16 (0.91–1.49)	1.09 (0.93–1.28)	1.18 (1.00–1.41)	1.67 (1.42–1.97)	< 0.0001	
Diabetes							0.80
No	1 [Reference]	1.09 (0.90–1.31)	1.12 (0.94–1.33)	1.18 (1.00–1.40)	1.66 (1.41–1.96)	< 0.0001	
Yes	1 [Reference]	1.04 (0.78–1.40)	1.21 (0.94–1.56)	1.21 (1.00–1.48)	1.51 (1.26–1.80)	< 0.0001	
Hyperlipidemia							0.43
No	1 [Reference]	1.26 (0.93–1.71)	1.26 (0.86–1.85)	1.47 (1.15–1.90)	1.82 (1.43–2.32)	< 0.0001	
Yes	1 [Reference]	1.05 (0.88–1.24)	1.13 (0.96–1.32)	1.13 (0.95–1.35)	1.56 (1.36–1.79)	< 0.0001	
Hypertension							0.67
No	1 [Reference]	1.21 (0.93–1.56)	1.21 (0.91–1.61)	1.25 (0.99–1.57)	1.76 (1.37–2.25)	< 0.001	
Yes	1 [Reference]	1.02 (0.81–1.27)	1.11 (0.93–1.34)	1.16 (0.99–1.36)	1.55 (1.36–1.77)	< 0.0001	
Stroke							0.07
No	1 [Reference]	1.09 (0.92–1.29)	1.13 (0.97–1.33)	1.17 (1.01–1.36)	1.68 (1.47–1.93)	< 0.0001	
Yes	1 [Reference]	1.00 (0.64–1.56)	1.18 (0.71–1.96)	1.28 (0.90–1.82)	1.26 (0.88–1.81)	0.100	
Waist, cm							0.12
< 100	1 [Reference]	1.06 (0.83–1.34)	1.19 (0.95–1.50)	1.33 (1.12–1.59)	1.80 (1.43–2.28)	< 0.0001	
≥ 100	1 [Reference]	1.11 (0.90–1.37)	1.11 (0.91–1.35)	1.09 (0.91–1.31)	1.51 (1.28–1.78)	< 0.0001	
Cardiovascular mortality^b^							
Age, years old							0.65
< 65	1 [Reference]	0.91 (0.45–1.85)	1.40 (0.86–2.29)	1.25 (0.68–2.30)	1.23 (0.72–2.13)	0.280	
≥ 65	1 [Reference]	1.20 (0.90–1.62)	1.21 (0.90–1.63)	1.17 (0.88–1.55)	1.75 (1.32–2.33)	0.001	
Sex							0.89
Female	1 [Reference]	1.06 (0.73–1.55)	1.25 (0.84–1.87)	1.11 (0.79–1.56)	1.48 (0.99–2.20)	0.07	
Male	1 [Reference]	1.29 (0.88–1.89)	1.23 (0.83–1.82)	1.30 (0.88–1.92)	1.88 (1.38–2.56)	< 0.001	
Ethnicity							0.32
White	1 [Reference]	1.26 (0.90–1.76)	1.18 (0.87–1.59)	1.13 (0.85–1.50)	1.72 (1.29–2.30)	0.002	
Black	1 [Reference]	1.08 (0.62–1.90)	1.73 (1.01–2.96)	1.48 (0.89–2.48)	1.97 (1.20–3.23)	0.010	
Mexican	1 [Reference]	0.56 (0.20- 1.59)	1.17 (0.46- 2.93)	1.49 (0.68- 3.27)	1.35 (0.57- 3.17)	0.270	
Other	1 [Reference]	1.08 (0.33–3.55)	1.93 (0.64–5.85)	1.94 (0.84–4.47)	2.17 (1.02–4.61)	0.030	
Education level							0.50
< High school diploma	1 [Reference]	1.02 (0.63–1.66)	1.32 (0.78–2.22)	1.14 (0.76–1.71)	1.80 (1.21–2.67)	0.010	
High school diploma	1 [Reference]	0.77 (0.42–1.38)	1.15 (0.70–1.90)	1.29 (0.83–2.00)	1.56 (0.91–2.69)	0.030	
> High school diploma	1 [Reference]	1.67 (1.09–2.55)	1.35 (0.89–2.04)	1.25 (0.79–1.98)	1.87 (1.28–2.72)	0.010	
Smoking status							0.07
Never	1 [Reference]	1.18 (0.85–1.64)	1.23 (0.86–1.78)	1.07 (0.75–1.52)	1.74 (1.18–2.56)	0.030	
Former	1 [Reference]	1.14 (0.76–1.72)	1.31 (0.85–2.01)	0.88 (0.60–1.31)	1.34 (0.98–1.83)	0.350	
Current	1 [Reference]	1.36 (0.55–3.39)	1.13 (0.49–2.62)	2.69 (1.20–6.02)	2.57 (1.34–4.92)	< 0.001	
Drinking status							0.29
Never	1 [Reference]	1.22 (0.62–2.40)	1.25 (0.72–2.19)	1.86 (1.02–3.39)	1.70 (1.00–2.88)	0.030	
Former	1 [Reference]	1.30 (0.80–2.11)	1.41 (0.93–2.14)	1.29 (0.86–1.93)	1.96 (1.32–2.93)	0.002	
Current	1 [Reference]	1.13 (0.74–1.72)	1.18 (0.72–1.94)	0.97 (0.65–1.44)	1.56 (1.07–2.29)	0.130	
Coronary heart disease							0.20
No	1 [Reference]	1.13 (0.84–1.52)	1.11 (0.80–1.54)	1.19 (0.92–1.55)	1.74 (1.30–2.32)	< 0.001	
Yes	1 [Reference]	1.41 (0.73–2.73)	1.97 (1.14–3.41)	1.28 (0.73–2.25)	1.65 (0.85–3.20)	0.140	
Congestive heart failure							0.31
No	1 [Reference]	1.09 (0.80–1.50)	1.25 (0.97–1.61)	1.15 (0.87–1.51)	1.60 (1.23–2.07)	< 0.001	
Yes	1 [Reference]	1.32 (0.59- 2.96)	1.55 (0.80- 3.00)	1.45 (0.84- 2.51)	2.23 (1.23- 4.04)	0.010	
Chronic kidney disease							0.35
No	1 [Reference]	1.02 (0.68–1.54)	1.23 (0.77–1.96)	1.11 (0.76–1.60)	1.27 (0.85–1.90)	0.220	
Yes	1 [Reference]	1.37 (0.96–1.95)	1.34 (0.99–1.81)	1.30 (0.99–1.71)	2.03 (1.47–2.81)	< 0.001	
Diabetes							0.39
No	1 [Reference]	1.37 (0.99–1.90)	1.26 (0.92–1.71)	1.18 (0.82–1.68)	1.81 (1.31–2.49)	0.004	
Yes	1 [Reference]	0.81 (0.45–1.46)	1.25 (0.83–1.88)	1.24 (0.88–1.73)	1.53 (1.09–2.15)	0.010	
Hyperlipidemia							0.61
No	1 [Reference]	1.33 (0.74–2.38)	0.87 (0.42–1.80)	1.41 (0.81–2.44)	1.48 (0.79–2.76)	0.190	
Yes	1 [Reference]	1.16 (0.83–1.61)	1.33 (0.99–1.77)	1.16 (0.89–1.52)	1.76 (1.36–2.28)	< 0.001	
Hypertension							0.65
No	1 [Reference]	1.58 (0.95–2.62)	1.31 (0.83–2.07)	1.34 (0.74–2.42)	1.63 (0.95–2.79)	0.130	
Yes	1 [Reference]	1.06 (0.73–1.54)	1.22 (0.91–1.64)	1.16 (0.89–1.51)	1.67 (1.25–2.23)	0.001	
Stroke							0.26
No	1 [Reference]	1.15 (0.85–1.57)	1.14 (0.87–1.51)	1.14 (0.88–1.47)	1.67 (1.29–2.17)	< 0.001	
Yes	1 [Reference]	1.99 (0.77–5.16)	2.65 (0.98–7.20)	2.36 (0.96–5.81)	2.75 (1.15–6.55)	0.010	
Waist, cm							0.45
< 100	1 [Reference]	1.26 (0.82–1.93)	1.28 (0.86–1.91)	1.36 (0.95–1.94)	2.24 (1.52–3.29)	< 0.001	
≥ 100	1 [Reference]	1.14 (0.80–1.62)	1.29 (0.91–1.83)	1.15 (0.76–1.72)	1.50 (1.08–2.10)	0.040	

HR, hazard ratio

^a^Measured by the nine-item center for the Patient Health Questionnaire (PHQ-9), and the range of PHQ-9 is between 0 and 27 with the highest score indicating the highest risk of depressive symptoms

^b^Model were all adjusted for age, sex, and ethnicity, education, smoking, drinking, waist, and systolic blood pressure, congestive heart failure, coronary heart disease, hypertension, hyperlipidemia, diabetes, chronic kidney disease, and stroke

## Discussion

We found both a linear and positive association between depressive symptom score with all-cause and CVD mortality from the NHANES database of 26,028 USA adults. Participants with depression had an increased risk of all-cause mortality and CVD mortality by 51% and 76%, respectively, compared with participants without depression. In addition, the presence of all nine depressive symptoms (uninterested in things, feeling down, sleep problems, tiredness, change of appetite, feeling bad about yourself, difficulty in concentration, change in speed of action, and suicidal ideation) in the PHQ-9 was independently associated with all-cause mortality, and the presence of six symptoms (except sleep problems, change of appetite, and suicidal ideation) was independently correlated with CVD mortality. Furthermore, there were significant interactions between age and drinking status on depression symptom score and all-cause mortality.

The association between major depression and mortality has been investigated in different populations from different countries [11, 25–30]. The Helsinki Birth Cohort Study followed 1995 participants in Finland for a mean duration of 14.1 years. Depression was diagnosed according to the Beck Depression Inventory, and increased mortality was observed as a result of depression (melancholic depressive disorder: adjusted HR, 1.49; 95% CI 1.02–2.20; non-melancholic depressive disorder: adjusted HR, 1.12; 95% CI 0.83–1.52) [31]. Another study of 24,542 participants aged 45–69 years in Central and Eastern Europe reported that an increase in depressive symptoms (“depressive symptoms” assessed by the Center for Epidemiologic Studies Depression Scale) was significantly and positively associated with CVD and all-cause mortality at a median follow-up of 7 years [32]. Meanwhile, a prospective study of 1999 community residents conducted in China with up to 12 years of follow-up discovered that time-dependent depression increased the risk of all-cause (adjusted HR: 1.48; 95% CI 1.26–1.73) and CVD death (adjusted HR: 1.40; 95% CI 1.08–1.82) by 48% and 40%, respectively [28]. Our results are in general agreement with previous studies. However, systematic reviews and meta-analyses have pointed out that this association may have been overestimated due to the large proportion of previous low-quality studies (such as small sample size, short follow-up time, and large cohort heterogeneity) [29, 33]. Considering that our study population was a nationally representative group of adults with a large sample size, the results of this study are highly credible.

Most previous studies classified populations as healthy or depressed by the sum of scale symptoms, ignoring the heterogeneity of depressive symptoms and important differences between individuals with depression [13, 34]. That is one of the main reasons for the slow progress and poor results in the field of clinical antidepressant development [35]. Until now, depression has been shown to be associated with all-cause mortality and cardiovascular mortality, but the association between a single depressive symptom and mortality has been unclear. This study found that all nine items of the PHQ-9 were independently associated with all-cause mortality, of which six were independent risk factors for CVD mortality. This finding provides new ideas for individualized treatment of patients with depression. Restricted activity was associated with chronic conditions, such as cerebrovascular accidents, cardiovascular diseases, and so on. This restricted activity associated with chronic disease appears to be significantly related to suicidal ideation [36]. What’s more, suicidal ideation also contributed largely to heart disease deaths [37]. Trouble sleeping or sleeping too much could increase incident CVD morbidity and mortality, especially among the elderly [38]. Poor appetite leads to poor nutritional status, which causes high mortality among hospitalized older patients [39]. Obesity from overeating reduces working capacity, decreases life quality, leads to high CVD morbidity [40], and causes early death [41, 42]. In addition, among older Chinese people, 2 individual symptoms (disturbed sleep and loneliness) were significantly associated with cardiovascular disease incidence [43]. Having little interest in doing things and Feeling tired or having little energy has been shown to be associated with all-cause mortality and cardiovascular mortality [44]. Importantly, although depressive symptoms tend to fluctuate over time, time-dependent depressive symptoms still increase the risk of all-cause mortality and cardiovascular mortality in the elderly [28]. However, our study found three specific depressive symptoms, including suicidal ideation, sleep problems, and changes in appetite, had no concern with CVD mortality, which might be where our results are inconsistent with previous studies. Three specific symptoms in our study were from PHQ-9 and were used to assess whether there was depression. Inconsistencies in symptom assessment methods might have contributed to the variable study results. Moreover, although the overall sample size of this study is large, the research specific to a certain depressive symptom weakens the sample size.

In the present study, subgroup analysis revealed an interaction between age and drinking status on the association between depressive symptoms scores and all-cause mortality. Compared to seniors, young and middle-aged people generally suffer from higher levels of stress due to various factors such as employment, marriage, and support for children and parents [45, 46]. Stress triggers increased inflammatory activity, leading to the onset and progression of depression [47]. In addition, elderly patients with depression usually have a combination of other chronic diseases and take more medications, some of which have the effect of inhibiting the inflammatory response. As a result, people with depression aged < 65 years have a higher risk of all-cause mortality compared to those aged ≥ 65 years. Drinking is related to an increased risk of premature death and physical and mental health problems in China [48]. The risk of symptoms of depression and anxiety is higher in abstainers and heavy drinkers [49]. Compared to moderate drinkers, the former and heavy drinkers continued to show increased risks of 51 and 45% for all-cause mortality in older adults, respectively [50]. Tom et al. revealed there were significant interactions between male drinkers and female ex-drinkers on the association between depressive symptoms and 11-year all-cause mortality [10]. This explained in part that there was high mortality in the former drinker with depression.

The association between depressive symptoms and increased risk of death is complex, with unhealthy lifestyle habits and pathophysiological changes being the focus of attention among many possible mechanisms. The depressed population often has poor lifestyle habits, such as smoking, excessive energy intake, overconsumption of alcohol, and a lack of exercise, among others [17, 51–54]. These unhealthy lifestyles have been recognized as risk factors for many chronic diseases such as CVD, metabolic diseases, and cancer, which further increase the risk of death [11, 55–57]. Similar results have been reported in several other studies [53, 58–60]. To sum up the above, quitting smoking, moderate alcohol consumption, and appropriate exercise may reduce the risk of death in individuals with depression. In terms of pathophysiology, depression increases the risk of death mainly through dysregulated inflammatory responses, oxidative stress damage, and hypothalamic–pituitary–adrenal (HPA) axis disorders. Higher levels of pro-inflammatory mediators, such as interleukin 6 (IL-6), C-reactive protein (CRP), and tumor necrosis factor⁃α (TNF-α), are usually detected in the blood of depressed patients compared to the healthy population [61]. Not only does inflammation play a key role in the development and progression of CVD (e.g., hypertension [62, 63], coronary atherosclerotic heart disease [64, 65], and heart failure [66]), but it is also closely related to other diseases such as diabetes, cancer, and the metabolic syndrome [14, 67–69]. In addition, inflammation-related factors activate cortisol secretion at three levels: the hypothalamus, pituitary, and adrenal cortex [70]. The HPA axis is hyperactive in depressed patients, resulting in a loss of circadian rhythm of cortisol secretion and a weakened ability to inhibit inflammatory control pathways, further producing more inflammatory responses and creating “positive feedback” [71].

## Strengths and limitations

The merits of this study include the national representativeness of the data, the large sample size, and the prospective design, which make the conclusions more convincing. In addition, we analyzed the associations of a single specific depressive symptom with outcomes (all-cause and CVD mortality), which is crucial. There are still some limitations to our study. Firstly, there were unadjusted residual confounding factors, such as marital status, depression-related medication use, etc. Secondly, causality could not be deduced since the study was an observational study. Thirdly, this study was conducted in the United States, and further validation is needed to see if the results could be generalized to other populations.

## Conclusions

The elevated depressive symptoms scores were strongly associated with an increased risk of all-cause and CVD mortality in USA adults. All nine items of the PHQ-9 were independently associated with all-cause mortality, of which six were independent risk factors for CVD mortality (trouble sleeping or sleeping too much, poor appetite or overeating, and suicidal ideation might not increase the risk of CVD mortality). These findings might be of significant value for the individualized treatment of patients with specific depressive symptoms.

### Supplementary Information

Additional file 1. The detailed description of missing data.

Additional file 2. Baseline characteristics according to depressive symptoms status in subpopulations of 21,552 participants.

Additional file 3. Association of depressive symptoms status with incidence of all-cause mortality and cardiovascular mortality in subpopulations of 21,552 participants.

## Data Availability

The data of the present study can be found here: https://www.cdc.gov/nchs/nhanes/index.htm.
