# Supplementary material for: Association of both depressive symptoms scores and specific depressive symptoms with all-cause and cardiovascular disease mortality
Source: Ann Gen Psychiatry. 2024 Jul 15;23:25. doi: 10.1186/s12991-024-00509-x (PMC11250981; doi:10.1186/s12991-024-00509-x)
Supplement: Supplementary file 1 — Additional file 1. The detailed description of missing data. [file 12991_2024_509_MOESM1_ESM.docx]

**Supplement material 1.** The detailed description of missing data.

| Characteristics | Missing number | Missing proportion |
| --- | --- | --- |
| Age, mean (SD), years | 0 | 0% |
| Female (%) | 0 | 0% |
| Ethnicity | 0 | 0% |
| Education level | 20 | 0.08% |
| Smoking status | 1334 | 5.13% |
| Drinking status | 1088 | 4.18% |
| Waist, mean (SD), cm | 753 | 2.89% |
| Systolic blood pressure | 571 | 2.19% |
| Diastolic blood pressure | 667 | 2.56% |
| Congestive heart failure | 1703 | 6.54% |
| Coronary heart disease | 1728 | 6.64% |
| Hypertension | 3 | 0.01% |
| Hyperlipidemia | 0 | 0% |
| Diabetes | 553 | 2.12% |
| Chronic kidney disease | 1295 | 4.98% |
| Stroke | 1668 | 6.41% |
