# Supplementary material for: Association of both depressive symptoms scores and specific depressive symptoms with all-cause and cardiovascular disease mortality
Source: Ann Gen Psychiatry. 2024 Jul 15;23:25. doi: 10.1186/s12991-024-00509-x (PMC11250981; doi:10.1186/s12991-024-00509-x)
Supplement: Supplementary file 2 — Additional file 2. Baseline characteristics according to depressive symptoms status in subpopulations of 21,552 participants. [file 12991_2024_509_MOESM2_ESM.docx]

**Supplement material 2.** Baseline Characteristics According to Depressive Symptoms Status in Sub-populations of 21,552 Participants.

|  | Participants, No. (%) | |  |  |
| --- | --- | --- | --- | --- |
|  | Overall | Depressive Symptoms | |  |
| Characteristics | N==21552 | No (N=19678) | Yes (N=1874)^a^ | *P*-value^b^ |
| Age, mean (SD), years | 49.38 (17.55) | 49.47 (17.72) | 48.47 (15.67) | 0.019 |
| Female (%) | 10646 (49.4) | 9456 (48.1) | 1190 (63.5) | <0.001 |
| Ethnicity |  |  |  | 0.029 |
| White | 10083 (46.8) | 9258 (47.0) | 825 (44.0) |  |
| Black | 4392 (20.4) | 3982 (20.2) | 410 (21.9) |  |
| Mexican | 3394 (15.7) | 3106 (15.8) | 288 (15.4) |  |
| Other | 3683 (17.1) | 3332 (16.9) | 351 (18.7) |  |
| Education level |  |  |  | <0.001 |
| <High school diploma | 5394 (25.0) | 4679 (23.8) | 715 (38.2) |  |
| High school diploma | 4962 (23.0) | 4522 (23.0) | 440 (23.5) |  |
| >High school diploma | 11196 (51.9) | 10477 (53.2) | 719 (38.4) |  |
| Smoking status |  |  |  | <0.001 |
| Never | 11583 (53.7) | 10843 (55.1) | 740 (39.5) |  |
| Former | 5331 (24.7) | 4917 (25.0) | 414 (22.1) |  |
| Current | 4638 (21.5) | 3918 (19.9) | 720 (38.4) |  |
| Drinking status |  |  |  | <0.001 |
| Never | 2881 (13.4) | 2633 (13.4) | 248 (13.2) |  |
| Former | 3971 (18.4) | 3520 (17.9) | 451 (24.1) |  |
| Current | 14700 (68.2) | 13525 (68.7) | 1175 (62.7) |  |
| Waist, mean (SD), cm | 99.03 (16.08) | 98.69 (15.85) | 102.55 (17.92) | <0.001 |
| Blood pressure, mean (SD), mmHg | |  |  |  |
| Systolic | 123.11 (18.21) | 123.15 (18.09) | 122.62 (19.42) | 0.222 |
| Diastolic | 70.11 (11.95) | 70.04 (11.91) | 70.84 (12.26) | 0.006 |
| History of comorbidities | |  |  |  |
| Congestive heart failure | 624 (2.9) | 501 (2.5) | 123 (6.6) | <0.001 |
| Coronary heart disease | 842 (3.9) | 725 (3.7) | 117 (6.2) | <0.001 |
| Hypertension | 9008 (41.8) | 8056 (40.9) | 952 (50.8) | <0.001 |
| Hyperlipidemia | 15568 (72.2) | 14118 (71.7) | 1450 (77.4) | <0.001 |
| Diabetes | 3818 (17.7) | 3347 (17.0) | 471 (25.1) | <0.001 |
| Chronic kidney disease | 3833 (17.8) | 3437 (17.5) | 396 (21.1) | <0.001 |
| Stroke | 775 (3.6) | 636 (3.2) | 139 (7.4) | <0.001 |

^a^ Defined as a score of 10 or higher on the nine-item center for the Patient Health Questionnaire in clinical studies.

^b^ *P*-value was based on T test or χ2.
