# Supplementary material for: Association of both depressive symptoms scores and specific depressive symptoms with all-cause and cardiovascular disease mortality
Source: Ann Gen Psychiatry. 2024 Jul 15;23:25. doi: 10.1186/s12991-024-00509-x (PMC11250981; doi:10.1186/s12991-024-00509-x)
Supplement: Supplementary file 3 — Additional file 3. Association of depressive symptoms status with incidence of all-cause mortality and cardiovascular mortality in subpopulations of 21,552 participants. [file 12991_2024_509_MOESM3_ESM.docx]

**Supplement material 3.** Association of depression with Incidence of all-cause mortality and cardiovascular mortality in Sub-populations of 21,552 Participants.

|  | HR (95% CI) |  |
| --- | --- | --- |
| Model | All-cause mortality | Cardiovascular mortality |
| Model 1^a^ | 2.04 (1.72-2.42) *** | 2.64 (1.97-3.54) *** |
| Model 2^b^ | 1.61 (1.35-1.92) *** | 2.11 (1.55-2.87) *** |
| Model 3^c^ | 1.42 (1.19-1.71) *** | 1.73 (1.27-2.35) *** |

^a^ Model 1: Age, sex, and ethnicity were adjusted.

^b^ Model 2: Model 1 plus education level, smoking status, drinking status, waist, and systolic blood pressure were adjusted.

^c^ Model 3: Model 2 plus congestive heart failure, coronary heart disease, hypertension, hyperlipidemia, diabetes, chronic kidney disease, and stroke were adjusted.

*, *P*-value<0.05; **, *P*-value<0.01; ***, *P*-value<0.001. Abbreviation: HR, hazard ratio.
